# Nonlinear optical physics at terahertz frequency

**DOI:** 10.1515/nanoph-2024-0109

**Published:** 2024-07-01

**Authors:** Yao Lu, Yibo Huang, Junkai Cheng, Ruobin Ma, Xitan Xu, Yijia Zang, Qiang Wu, Jingjun Xu

**Affiliations:** 12538 Nankai University, Tianjin, China

**Keywords:** terahertz, nonlinear optics, light–matter interaction

## Abstract

Terahertz (THz) waves have exhibited promising prospects in 6G/7G communications, sensing, nondestructive detection, material modulation, and biomedical applications. With the development of high-power THz sources, more and more nonlinear optical effects at THz frequency and THz-induced nonlinear optical phenomena are investigated. These studies not only show a clear physics picture of electrons, ions, and molecules but also provide many novel applications in sensing, imaging, communications, and aerospace. Here, we review recent developments in THz nonlinear physics and THz-induced nonlinear optical phenomena. This review provides an overview and illustrates examples of how to achieve strong THz nonlinear phenomena and how to use THz waves to achieve nonlinear material modulation.

## 1 Introduction

Light–matter interaction (LMI) has been a long-discussed topic in optics and condensed-matter physics [1]. In the early studies, LMI at visible and infrared frequencies were mainly discussed since lasers at such frequencies are highly developed. LMI indicates how light can polarize, heat, melt, and ablate the electrons, ions, or molecules within most materials. However, the significant difference in mass of these microparticles causes an individual energy scale. By considering the resonant frequencies of these different particles, we find that each particle can easily interact with light at a similar frequency but is minimally affected by light far away from the resonant frequency. The Lorentz oscillator equation and energy band theory provide the fundamental basis for investigating traditional LMI that involves high frequency light.

**Figure 1:**
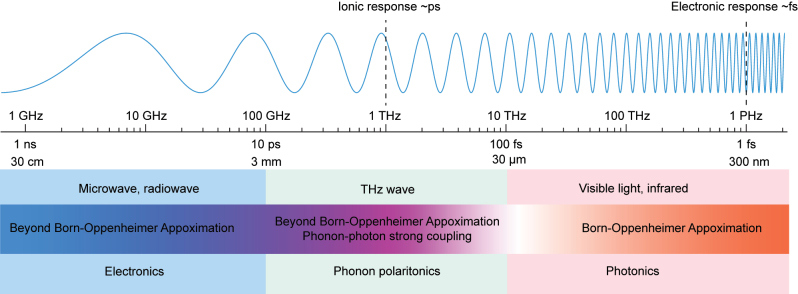
Dominant mechanisms in the light–matter interaction processes during various frequencies in bulk polar crystals.

For LMIs between high frequency light and crystal solids, the heavy ions are less possibly to contribute to such fast oscillations. Therefore, the traditional approaches assume that the ions are fixed in the crystal, only electrons can respond to such fast electromagnetic oscillations. The motion of the ions is usually taken as a perturbation to the electronic polarizations, which is known as Born–Oppenheimer approximation [2], [3]. Nevertheless, when the pump beam becomes low-frequency light such as terahertz (THz) or microwave, the ionic polarization would dominate the whole LMI process. In this case, Born–Oppenheimer approximation is not valid anymore. Especially, for the THz pump light (about 0.1∼10 THz), at which the optical phonon frequency for majority crystal materials is located, strong coupling between the input THz waves and the optical phonons forms a new quasiparticle, stimulated phonon polaritons, which plays unneglected role in LMI at the THz frequency and also causes large THz nonlinearity in polar crystal solids [4]. In Figure 1, we show the dominated LMI mechanisms through various wavelengths. Notably, some physical processes, including Raman/Brillouin scattering, although the pump light could be at visible or infrared, are beyond Born–Oppenheimer approximations.

Besides, many low-dimensional materials, such as graphene, are also found to behave very large THz nonlinearity due to its Dirac electrons at the special band structures. Metamaterials/metasurfaces, plasmons, and superconductors have also been options to achieve THz nonlinearity.

In this review, we report some states of the art of the nonlinear LMI process at THz frequency. A series of nonlinear phenomena at THz frequency or induced by THz waves in recent years are displayed, including frequency conversion, Kerr effect, nonlinear absorptions, as well as THz modulating ions, molecules, and electrons in materials to realize different nonlinear phenomena. In Section 2, we introduce the nonlinear LMI theory when involving THz waves, such as electronic and ionic Lorentz–Drude model, nonlinear Huang equations, and hot Dirac electrons in graphene, and try to explain how THz nonlinearity arises. In Section 3, we review the THz frequency conversion studies through nonlinear optics, such as second-harmonic generation, sum/difference-frequency generation, third-harmonic generation, high-harmonic generation, and parametric amplification processes. In Section 4, recent progress on THz Kerr effect and some derivative phenomena is reported. In Section 5, we review the THz nonlinear absorption processes including saturate absorption and multiphoton absorption. In Section 6, we review the THz-induced nonlinearity at visible or infrared frequencies. In Section 7, we summarized all the development and talked about the possible progress on THz nonlinearity in the near future.

## 2 Theory of nonlinear light–matter interactions

Nonlinear optics originates from the nonlinear polarization of materials by light, which means slight relative shift of positive and negative electric charge in opposite directions within a material induced by an external electric field. Electronic polarization occurs when an electric field distorts the negative cloud of electrons around positive atomic nuclei in a direction opposite the field. Ionic polarization requires an electric field to move the positive and negative ions along the opposite directions. This slight separation of charge makes one side of the atom somewhat positive and the opposite side somewhat negative. In some materials whose molecules are permanently polarized by chemical forces, such as water molecules, some of the polarization is caused by molecules rotating into the same alignment under the influence of the applied electric field.

### 2.1 Electronic nonlinearity and ionic nonlinearity

In linear optics, the polarization is very slight such that the restoring force is linearly changed with the particle displacement. However, for crystal solid bound by either ionic or covalent bonds, the potential of each individual ion, the Lennard-Jones potential or Madelung energy [5], is highly nonquadratic. Instead, the interaction between different electrons is much weaker. In this case, the single-electron approximation is usually used to consider the electronic polarization as a very simple Lorentz-oscillator model, which considered an oscillator between an electron and an atomic nucleus. Similarly, the ionic nonlinearity can be considered as a Lorentz oscillator between a positive ion and a negative ion.

For the nonlinear LMI involving visible or infrared light, only the electronic nonlinearity is contributed since the ions are too heavy to respond such fast oscillations. Here, the ions are assumed to be fixed in the lattice according to the Born–Oppenheimer approximation. The Lorentz anharmonic oscillation equation reads [6].

(1) $${\ddot{x}}_{e}+\gamma_{e}{\dot{x}}_{e}+\omega_{0e}^{2}x_{e}=-\alpha_{e}x_{e}^{2}+E_{\text{ex}}\left(e/m_{e}\right),$$

where *x*_*e*_ and *ω*_0*e*_ represent the electron displacement and eigenfrequency of the electron–ion oscillator, *γ*_*e*_ indicates the corresponding damping rate, and *α*_*e*_ describe the primary anharmonic coefficient. *E*_ex_(*e*/*m*_*e*_) describes the driving force that the input light field exert on electrons. Importantly, when considering the centrosymmetric materials, the even-odder nonlinearity is canceled due to structure symmetry, and thus the primary nonlinear restoring force should be $-\beta_{e}x_{e}^{3}$ rather than $-\alpha_{e}x_{e}^{2}$ .

If the nonlinear LMI involving low-frequency light, such as microwave frequency, it is more appropriate to consider ionic nonlinearity by the anharmonic oscillator equation of the positive–negative ions [7].

(2) $${\ddot{x}}_{i}+\gamma_{i}{\dot{x}}_{i}+\omega_{0i}^{2}x_{i}=-\alpha_{i}x_{i}^{2}+E_{\text{ex}}\left(q/m\right).$$

This equation is just a simple transplant from Eq. (1), but all parameters are changed from electron–ion oscillator to positive–negative ions oscillator. Differently, *x*_*i*_ indicates a reduced displacement that *x*_*i*_ = (*x*_+_ *x*_−_)/(*x*_+_ + *x*_−_), with *x*_+_ and *x*_−_ are displacements of positive and negative ions, respectively. Similarly, $-\alpha_{i}x_{i}^{2}$ should be replaced by $-\beta_{i}x_{i}^{3}$ when centrosymmetric crystals are considered. In some papers, LMIs involving THz wave are consider to subordinate to Eq. (2) [7]. However, Eq. (2) does not consider the coupling term of THz wave and optical phonons, so we don’t think the ionic nonlinearity provides a general model to describe the nonlinear LMI at THz frequency.

### 2.2 Stimulated phonon polariton and THz wave nonlinearity

THz waves, at which the optical phonon frequency for majority crystal materials is located, would have a strong coupling between optical phonons in ionic crystal. Physically, when the external driving frequency is near from the resonant frequency of optical phonons, the displacements of ions are largely increased, so nonlinear polarization becomes far more significant. Considering the forced ionic oscillations by input THz waves would radiate another THz wave that driving the successive ions, a new LMI model through the stimulated phonon polariton is obtained. This mechanism is mathematically described by nonlinear Huang equations [4]:

(3) $$\left\{\begin{array}{c} \ddot{x}+\gamma \dot{x}+\omega_{0}^{2}x={\left(Nm\right)}^{-1/2}bE-\alpha x^{2}+E_{\text{ex}}\left(q/m\right).\;\\ P={\left(Nm\right)}^{1/2}bx+\epsilon_{0}\left(\varepsilon_{\infty }-1\right)E.)\; \end{array}\right.$$

In nonlinear Huang equations, the contribution of stimulated phonon polaritons is considered. Compared with the anharmonic oscillator equation of ions (Eq. (2)), the cross contribution of macroscopic electric field and ionic displacement are added, where the coefficient $b=\omega_{0}\sqrt{\epsilon_{0}\left(\varepsilon_{0}-\varepsilon_{\infty }\right)}$ indicates their coupling, with *ɛ*_0_ and *ɛ*_∞_ are the relative permittivity at high and low frequencies, respectively. *ϵ*_0_ = 8.854 × 10^−12^ F/m represents the vacuum permittivity. Similarly, −*αx* ^2^ should be replaced by −*βx* ^3^ when centrosymmetric crystals are considered.

**Figure 2:**
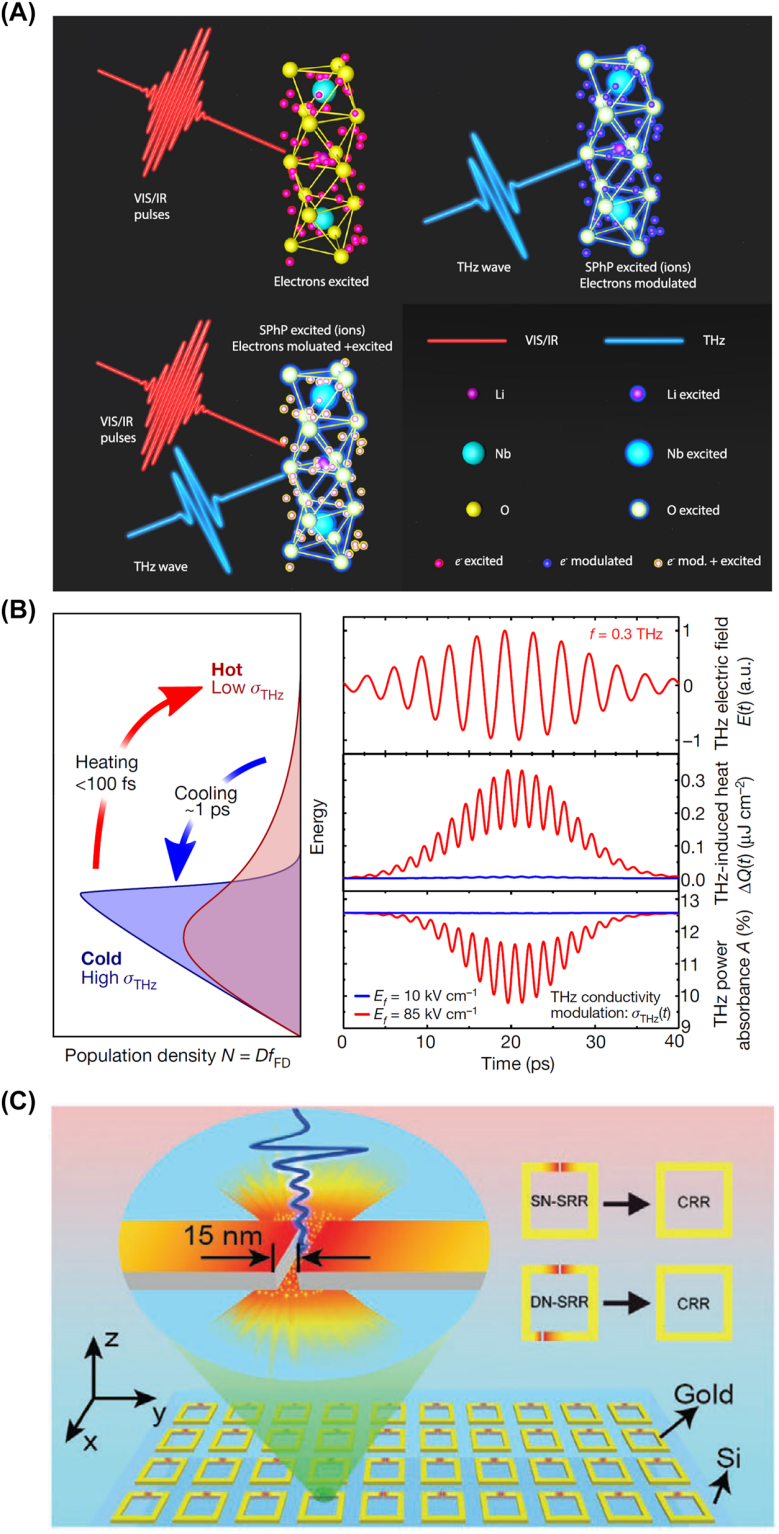
Approaches to achieve strong THz nonlinearity. (A) Stimulated phonon polaritons mediated light–matter interaction mechanism in lithium niobate crystals [4]; (B) strong THz nonlinearity induced by Dirac fermions in graphene [9]; (C) metal plasmon-enhanced strong THz nonlinearity [10]. Reprint permission obtained for references [4], [9], [10].

Nonlinear Huang equations (Eq. (3)) describe a new nonlinear LMI at THz frequency. As Figure 2(A) shows, the classic LMI at visible or infrared frequency light only takes place between light and electrons protected by Born–Oppenheimer approximation; the LMI at THz frequency directly excite stimulated phonon polaritons, which are described by Eq. (3); stimulated phonon polaritons excited by THz waves would change the LMI mechanism at high frequency by stimulated phonon–polariton–electron coupling. Through this mechanism, the nonlinear polarizations at THz frequency can be greatly enhanced [8].

Graphene is also one of the best candidates to show exceptional nonlinear optical properties at THz frequency range [11]. The origins of the large nonlinearity of graphene come from its particular band structures. The linear dispersion of electrons near the Dirac point makes the graphene electron behave like a zero-mass fermion, namely Dirac fermion, which is different from the bulk materials. In thermal equilibrium, the intraband conductivity of graphene is determined by the carrier concentration and the temperature of background electronic population. When excited by an external THz field, the electronic population remains constant, and thus the electronic temperature rise would cause a conductivity reduction. Therefore, a THz current is induced to deposit energy into the electronic population. Since the mobility of the Dirac electrons is large enough to transfer the instantaneous current to the thermal temperature of the background electron population. Therefore, the conductivity of graphene is correspondingly decreased [9].

As a result of these interdependencies, the THz conductivity of graphene depends strongly nonlinearly on the driving THz field, the stronger the field, the smaller the conductivity becomes. The cooling of the hot Dirac electrons is mediated by phonon emission with THz rate. The heating and cooling processes of background Dirac electron population induce a reduction and recovery of the intraband THz conductivity in graphene, as shown in Figure 2(B). Such a THz field–induced nonlinear modulation of the THz conductivity results in THz period absorption change, resulting nonlinear radiations with respect to the input THz waves [9].

Metasurfaces also provide a strong localization and field enhancement to the THz waves, which naturally could arise THz nonlinear responses [10]. A typical THz metasurface structure is shown in Figure 2(C), in which the THz waves are strongly localized at the nanogap between the metal split ring resonators by plasmonic resonance. This approach obtains an observable nonlinearity through simply raising the pump THz field.

Actually, the mentioned three mechanisms are different but not contradicted to one another. From the materials, stimulated phonon polaritons only exist in polar crystals but not in single-atom materials like graphene, silicon. The hot Dirac fermions only work in low-dimensional materials, such as graphene. Differently, the local plasmon resonance on metasurface directly works on enhancing the pump light, which can be used together with the other two mechanisms to achieve far stronger THz nonlinearity.

## 3 Nonlinear THz frequency conversion

The ability to realize the nonlinear optics frequency conversion is important to a wide range of technologies, such as material characterization, ultrashort pulse measurements, optical information processing, telecommunications, and imaging spectroscopy. Recently, the nonlinear optical frequency conversion of THz wave has emerged as a powerful platform for controlling and manipulating solid-state materials, and especially complex condensed matter systems with strongly correlated electrons. Especially, the investigation of enhancing THz nonlinear frequency conversion effects is particularly significant, due to these nonlinear mechanisms could provide potential values on the development and design of new reconfigurable planar THz nonlinear devices, and the study of classical and quantum phase transitions.

### 3.1 Second-order THz nonlinear phenomena

Second-order nonlinear processes at THz field, for example, THz second-harmonic generation (SHG), have been observed in a few early pioneering studies. Mayer and Keilmann used gallium arsenide (GaAs) and lithium tantalate (LiTaO_3_) with intrinsic second-order susceptibilities, which originate from lattice resonances, and first obtained THz SHG in the experiment [12]. THz SHGs were subsequently investigated in artificially structured quantum wells, where asymmetric electron responds to the THz field direction [13], [14], [15]. In these investigations, the asymmetric electron responses result from the frequency modulation of a damped Bloch oscillation with an external field or the asymmetric design of quantum wells. However, the SHG conversion efficiencies in these reported studies are unavoidably low, because the inherent nonlinearities of crystalline materials are low or the couplings of THz waves and nanoscale quantum well structures are limited. Furthermore, it is important to observe SHGs by limiting the operating frequency band to the lattice resonance frequencies of GaAs and LiTaO_3_ and sophisticatedly engineer potential wells [13], [14], [15]. THz SHG can still be observed in those media with an applied electric bias, which called electric field–induced SHG (EFISHG) [16], [17]. Further improvement of the conversion efficiency is also required for this platform in practical applications.

**Figure 3:**
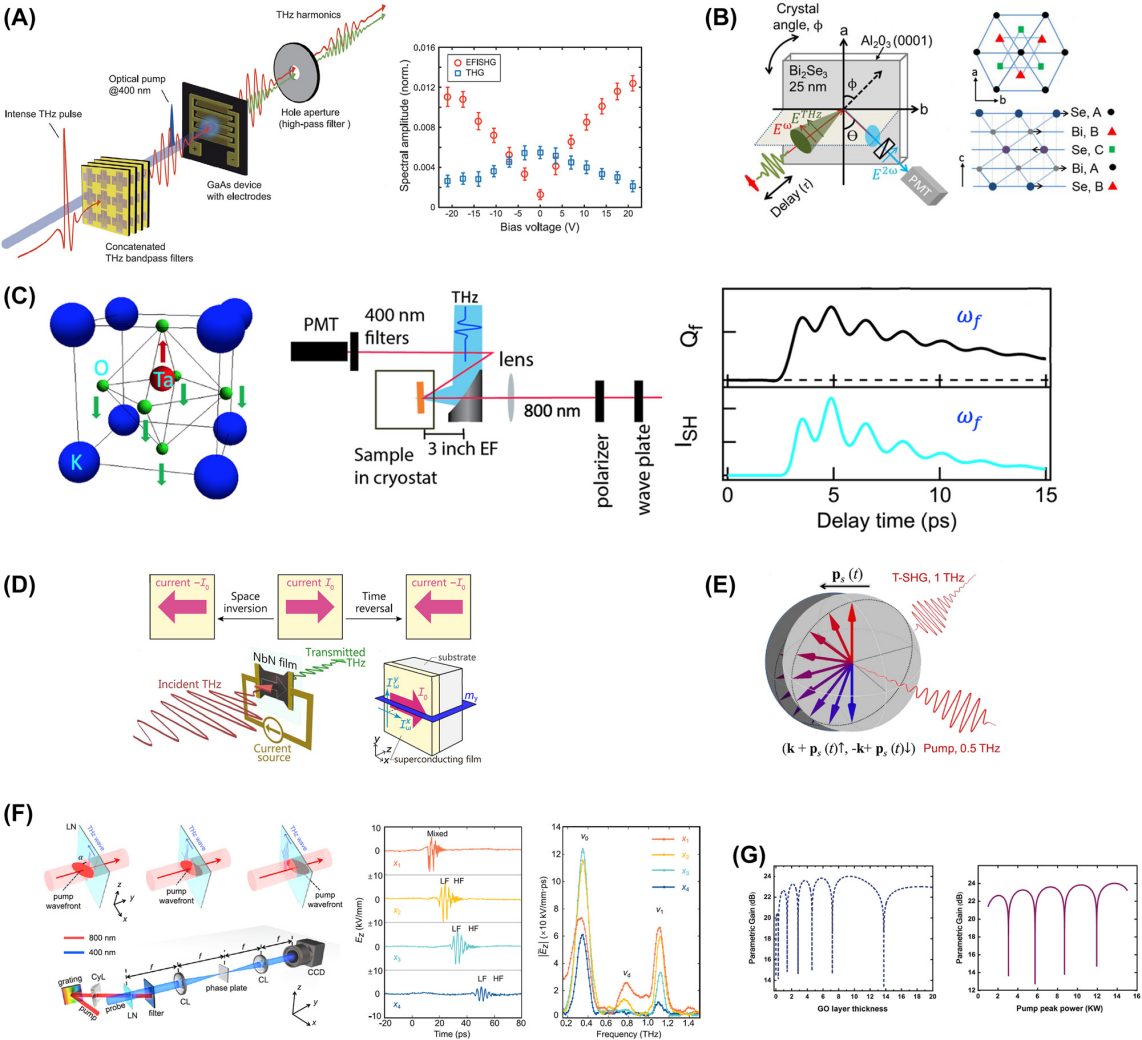
Approaches to achieve strong THz second-harmonic generation (SHG). (A) Experimental schematic of THz harmonic generation. Measured EFISH (red circle) and third-harmonic (blue square) amplitudes as a function of bias voltage. The error bars denote the standard deviations of each measurement [18]; (B) setup for THz pump, SHG-probe experiments, and phonon properties of Bi_2_Se_3_. The *p*-polarized THz excitation pulse (*E* ^THz^) is incident on the sample at *θ* = 30°. A collinear, copolarized 1.55 eV probe pulse (*E* ^*ω*^) arrives at a delay *τ* after the THz pulse. The *p*-polarized reflected SHG signal, *E* ^2*ω*^, at 2*ω* = 3.1 eV, is collected with a photomultiplier tube. The crystal is rotated about the azimuthal angle *ϕ* to measure the static SHG symmetry (when blocking the THz pump pulse) or the symmetry of the THz-induced changes. Schematic of the crystal structure of a quintuple layer of Bi_2_Se_3_ in the *ab* plane and a side view along the *c*-axis; the arrows illustrate the lattice displacements associated with the phonon [19]; (C) cubic lattice structure and ferroelectric soft mode of KTaO_3_ (left). Schematic illustration of the THz pump SHG probe setup (middle). PMT, photomultiplier tube; EF, effective focal length. Illustration of possible soft-mode displacement *Q*_*f*_ and THz-induced SHG signal *I*_SH_ as a function of delay time in a THz-driven transient ferroelectric phase (right) [20]; (D) DC supercurrent *I*_0_ indicated by the arrow changes the direction under each time-reversal and space-inversion operations. Schematic view of the THz transmittance experiments under the DC supercurrent [21]; (E) SHG by THz wave acceleration of superfluid momentum [22]; (F) experimental setup and illustration of velocity matching between pump pulse and generated THz wave (left). Evolution of the THz field with time at different positions and their corresponding Fourier spectra (right) [8]; (G) parametric efficiency versus the thickness of graphene oxide (GO) layer (left) and pump peak power (right) [23]. Reprint permission obtained for references [8], [18], [19], [20], [21], [22], [23]. (B) Reproduced with permission [19], Optica Publishing Group. (G) Reproduced with permission [23], Optica Publishing Group.

Recent studies have reported to overcome these limitations, and effectively improve the conversion efficiencies of THz SHG, as shown in Figure 3. The THz EFISHG can be substantially enhanced by utilizing the large third-order nonlinearity from the intervalley scattering in photoexcited GaAs [18], compared with those from crystals with ionic resonances or nanostructured semiconductors. A brief overview of the THz EFISHG experiments is shown in Figure 3(A), and corresponding amplitude of the EFISH wave increased with the magnitude of the bias voltage increased until saturation was reached, as shown in Figure 3(A). Figure 3(B) shows a unique approach to modulate light–matter interactions in THz field by combining resonant excitation in the topological insulator Bi_2_Se_3_ with optical SHG [19]. SHG is used to separate the resulting symmetry changes at the surface from the bulk. The lattice vibrations with a pair of THz pulses are also coherently controlled, as shown in Figure 3(B). In quantum materials, light-induced ferroelectricity in quantum paraelectrics is a new way to achieve dynamic stabilization of hidden orders. THz pump SHG probe is used to investigate the quantum paraelectric KTaO_3_ [20], as shown in Figure 3(C). The possibility of driving a quantum paraelectric KTaO_3_ into a transient ferroelectric phase is proved, as depicted in Figure 3(C). The nonreciprocal electromagnetic responses in s-wave superconductors at THz under DC supercurrent bias are also studied [21], as shown in Figure 3(D). Furthermore, it was also reported THz light-induced SHG in the platform of superconductors with inversion symmetry, which forbids the even-order nonlinearities [22], as shown in Figure 3(E).

During the nonlinear frequency conversion, difference-frequency generation (DFG) also plays an important role. Figure 3(F) shows a giant second-order nonlinear susceptibility at THz frequency, where both the input and output signals THz waves [8]. Moreover, the nonlinearity enhancement mainly resulted from the stimulated phonon polaritons rather than merely from the ionic nonlinearities. The giant second-order nonlinearity for DFG is obtained through a phase-matching configuration when judicious designing the waveguide dispersion. Figure 3(F) shows the experimental setup and illustration of velocity matching between pump pulse and generated THz wave, and the evolution of the THz optical field with time at different positions and their corresponding Fourier spectra are shown in Figure 3(F).

Recent studies also revealed a widely tunable regenerative THz parametric amplifier based on degenerate four-wave mixing in an asynchronously pumped highly nonlinear medium. The output power, the tuning frequency range, and the power conversion efficiency of THz waves could be considerably improved in the proposed design [23], as shown in Figure 3(G).

### 3.2 Third-harmonic generation of THz wave

**Figure 4:**
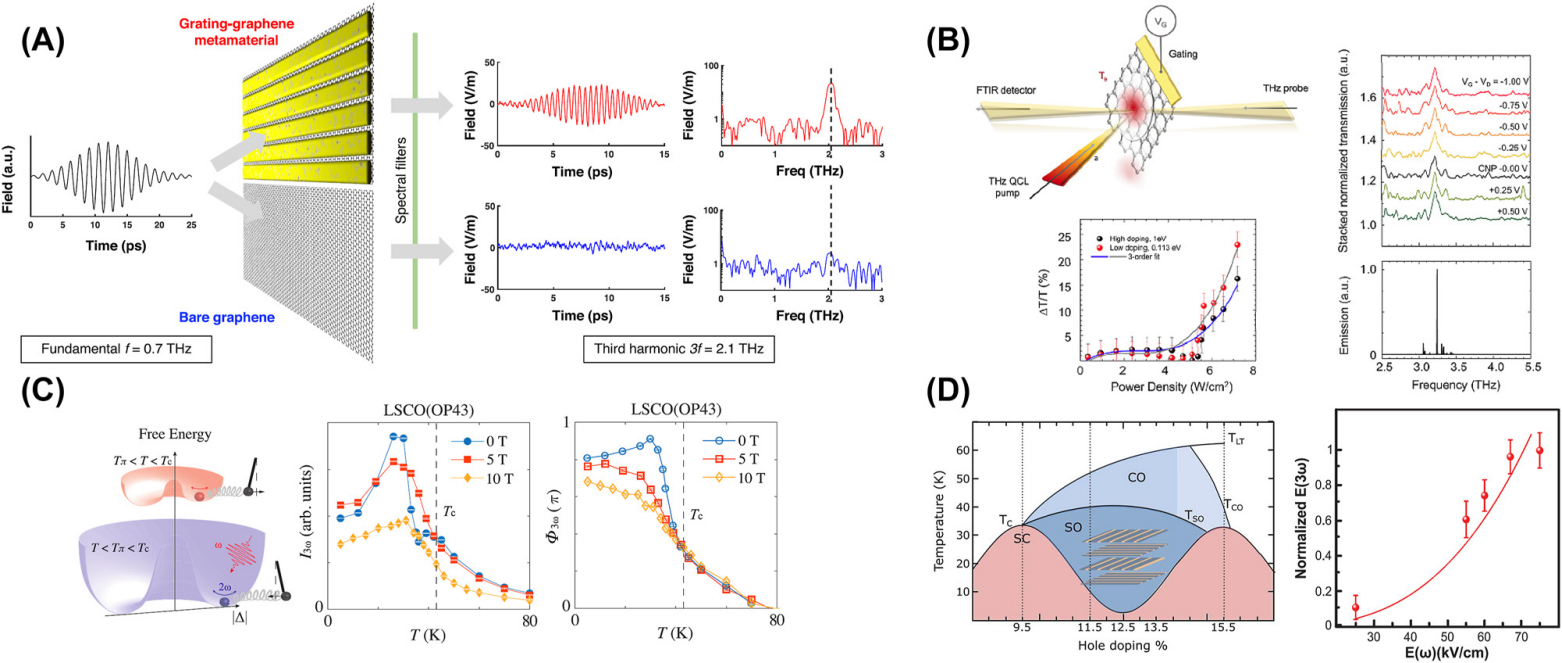
Approaches to enhance THz third-harmonic generation (THG). (A) Schematics and results of THz third-harmonic generation (THG) in grating-graphene metamaterial [24]; (B) schematic diagram of the THz pump and probe measurement (left-top panel). Stacked transmission curves retrieved from the ratio of the experimental transmissions measured on single-layer graphene (left-bottom panel). FTIR emission spectrum of the single-plasmon QCL employed as a pumping source, and experimental transmission change (right) [25]; (C) illustration of the amplitude oscillation of the superconducting order parameter (2*ω*) driven by electromagnetic radiation (*ω*) and its coupling to another collective mode (black pendulum) at temperatures above and below *T*_*π*_, i.e., the antiresonance temperature. Temperature dependence of *I*_3*ω*_ with a magnetic field applied along the *c*-axis of *x* = 0.16 (OP43). Corresponding temperature dependence of Φ_3*ω*_ from the sample (OP43) [27]; (D) phase diagram for La_2−*x*_Ba_*x*_CuO_4_. SC, SO, and CO denote the bulk superconducting, spin- and charge-ordered (striped), and charge-only ordered phases, respectively (left). *T*_C_, *T*_SO_, and *T*_CO_ are the corresponding ordering temperatures. *T*_LT_ denotes the orthorhombic-to-tetragonal structural transition temperature. Electric field dependence of the third-harmonic amplitude for *T* = 5 K (right) [28]. Reprint permission obtained for references [24], [25], [27], [28].

Moreover, THz third-harmonic generation (THG) has also received a burgeoning amount of interest in the field of THz nonlinear processes. Inherently minimal light–matter interaction length in two-dimensional materials is a serious challenge of limiting the overall THG conversion efficiency, which was overcome in the recent work [24]. The metamaterial platform combines graphene with a photonic grating structure, as shown in Figure 4(A). The field enhancements of the fundamental and third-harmonic frequencies for the grating-graphene metamaterial are shown in Figure 4(A), which both significantly enhance compared with those of bore graphene in Figure 4(A), especially the nonlinearity of 50 times larger. The THG conversion efficiency can be also improved by the utilization of the single-layer graphene (SLG), due to its highly nonlinear material (*χ* ^(3)^ ∼ 10^−9^ m^2^/V^2^ in the far-infrared). The nonlinear response of SLG can be controlled using an ionic liquid gate that tunes the graphene Fermi energy up to > 1.2 eV [25], as shown in Figure 4(B). Furthermore, except for the graphene, Dirac materials are another quantum materials to effectively possess high THz THG conversion efficiency. The strong THz THG nonlinearity in 2D topological insulators based on single HgTe/CdTe quantum well has been recently reported [26].

Except for metamaterials, superconductors are also utilized to enhance the THG conversion efficiency, for example, cuprate high-*T*_*c*_ superconductors, which are known for the coexistence of competing orders and their intertwined interactions [27]. The Fano resonance is a typical spectroscopic signature of the interaction between a discrete mode and a continuum of excitations, which is characterized by the asymmetric light-scattering amplitude of the discrete mode as a function of the electromagnetic driving frequency. A new type of Fano resonance manifested by the THz THG response of cuprate high-*T*_*c*_ superconductors has been reported, as depicted in Figure 4(C), where both the amplitude and phase signatures of the Fano resonance are resolved [27], as shown in Figure 4(C). Furthermore, unconventional superconductivity in the cuprates coexists with other types of electronic order, some of which are invisible to most experimental probes due to their symmetry. For example, superfluid stripes are not easily validated with linear optics because of the stripe alignment causing interlayer superconducting tunneling to vanish on average. It has been shown that this limitation is overcome in the nonlinear optical response with a giant THz THG, characteristic of nonlinear Josephson tunneling [7], [28], [29], [30], [31], [32], as shown in Figure 4(D).

### 3.3 High-order THz nonlinear optics

**Figure 5:**
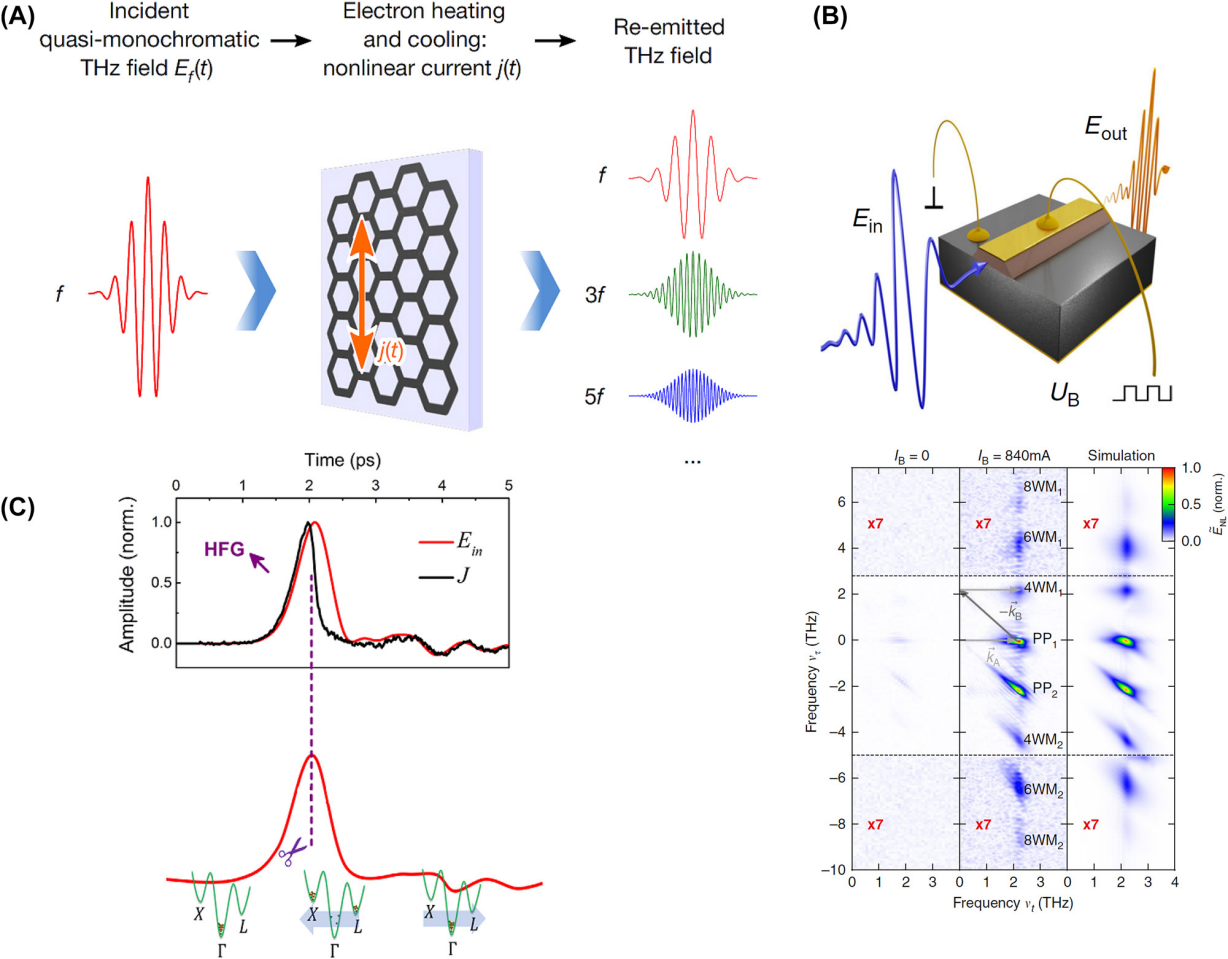
Approaches to generate or enhance THz high-order harmonic generation (HHG). (A) Schematic of the experiment: quasi-monochromatic, linearly polarized THz pump wave from the THz source is incident on a graphene sample, single-layer CVD-grown graphene deposited on SiO_2_ substrate [9]; (B) experimental arrangement (top); THz waveform *E*_in_ (blue) is focused onto the QCL facet and the transmitted waveform (*E*_out_, orange) is detected electro-optically. The rectangular modulation of the bias voltage allows the current-induced change of the nonlinear response to be extracted. The bottom panel shows 2D-amplitude spectrum ${\tilde{E}}_{\text{NL}}\left(\nu_{t},\nu_{\tau }\right)$ of the nonlinear response (*I*_B_ = 0 and 840 mA), as well as the corresponding simulated 2D-amplitude spectrum of the nonlinear response (*I*_B_ = 840 mA) [35]; (C) incident THz waveform (*E*_in_) and the calculated current density (*J*) with the field strength of 190 kV/cm (top). Schematic of the experiment in momentum space (bottom): intense THz transient induces carrier intervalley scattering. The increased field strength leads to a damping of the subcycle current density and an increase in the total relaxation time [36]. Reprint permission obtained for references [9], [35], [36].

Multiple optical harmonic generation, the multiplication of photon energy due to the nonlinear interaction between light and matter, is a key technology in modern optoelectronics and electronics, because it converses the optical or electronic signals of the fundamental frequencies into signals with much higher frequency, except for SHG and THG, and allows the generation of frequency combs. The generation of THz harmonics up to the seventh order in single-layer graphene at room temperature and under ambient conditions was reported [9], as shown in Figure 5(A), which is driven by THz fields of only tens of kilovolts per centimeter, and with conversion efficiencies in the order of 10^−3^, 10^−4^, and 10^−5^ for the third, fifth, and seventh harmonics generation, respectively. At room temperature, THz-field driven high-harmonic generation in the three-dimensional Dirac semimetal Cd_3_As_2_ was also reported, which was excited by linearly polarized multicycle THz pulses, and the conversion efficiency of the third, fifth, and seventh harmonics is very high and was detected via time-resolved spectroscopic technique [33].

Furthermore, many important excitations of solids are found at low energies; thus, much can be gained from the extension of nonlinear optics to mid-infrared and THz frequencies. For example, the nonlinear excitation of lattice vibrations has enabled the dynamic control of material functions. However, it has only exploited seventh-harmonic generation at THz field strengths near one million volts per centimeter. Recently, it has achieved an order-of-magnitude enhancement of the field strength and explored higher-order phonon nonlinearities of fifth-harmonic generation [34].

The significant subcycle dynamics have been notoriously difficult to access in operational THz quantum cascade lasers (QCLs), although the exploitation of ultrafast electron dynamics in QCLs holds enormous potential for intense. In view of this problem, high-field THz pulses to perform the first ultrafast two-dimensional spectroscopy of a free-running THz QCL has been employed, as shown in Figure 5(B), and strong incoherent and coherent nonlinearities up to eight-wave mixing are detected below and above the laser threshold [35], as shown in Figure 5(B). Nonlinear optical effects induced by intense were also investigated, and the recent work reported a truncation of the half-cycle THz pulse and an emission of high-frequency THz photons [36], as shown in Figure 5(C).

Besides, there are also some other investigations on high-order THz nonlinear phenomena such as four-wave mixing [37], [38] and six-wave mixing [39], which provides insights to the progress of the THz nonlinearities.

## 4 THz Kerr effect

Kerr effect, a kind of third-order nonlinear effect, is a degenerate case of the four-wave mixing effect, which manifests that the refractive index depends on the light intensity, lying at the heart of many optical applications. For visible and near-infrared light, the Kerr coefficient is generally weak and considered to mainly originate from the electronic nonlinearity. The rotation, orientation, and arrangement of molecules, as well as lattice vibrations, can respond to THz waves, which mean the ionic nonlinearity can be triggered to achieve giant Kerr nonlinearity in the THz regime. In this section, we show the observation of the THz Kerr effect (TKE), the measurement of the coefficients and the origins, as well as the refractive index at optical frequencies perturbed by THz pulses.

### 4.1 Refractive index change via THz Kerr effect

At present, measuring the Kerr coefficient of different substances is one extremely important frontier of TKE. So far, the observation of TKE and the measurement of the coefficients are mainly based on THz time-domain spectroscopy (THz-TDS) systems or *z*-scan technique. Actually, it is difficult to distinguish the TKE probed by optical light and THz-induced Kerr effect, and there are some overlaps between the two effects in the literature [40], [41]. Thus, we just give a brief overview here.

**Figure 6:**
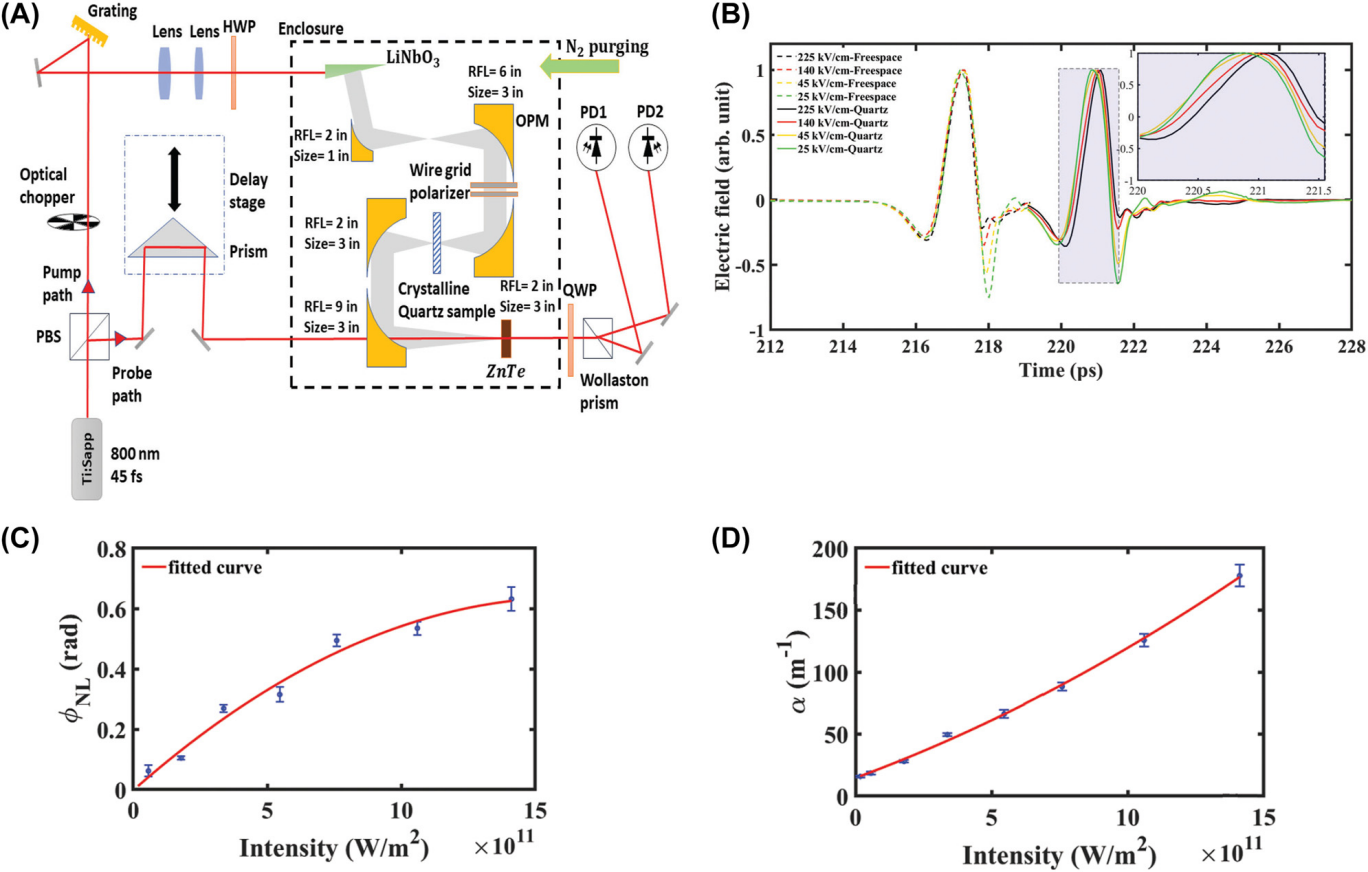
Strong Kerr response in quartz [43]. (A) The schematic of THz-TDS experiments; (B) THz time-domain signals in free space (dashed lines) and crystalline quartz (solid lines) for different signal levels. (C) Nonlinear phase experienced by the signal of different amplitudes at 0.4 THz. (D) Absorption coefficient for each signal level at 0.4 THz. Reprint permission obtained for references [43].

It has been predicted that crystals would exhibit extremely large nonlinear refractive indices at THz frequencies due to lattice vibration, stimulated phonon polaritons, or rotation/rearrangement of molecules, which can exceed the value of the nonlinear refractive index in the visible and near-IR ranges by several orders of magnitude [4], [42]. Then, the prediction was confirmed, taking quartz as an example. A very strong nonlinear response in crystalline quartz in the THz frequency was experimentally observed through THz-TDS [43]. As depicted in Figure 6(A), a beam of laser is split into the pump and probe paths, where the pump beam is used to generate intense THz pulses in an optical rectification process in lithium niobate (LiNbO_3_) using the pulse-front tilting technique. The THz pulses pass through the sample first and then through the ZnTe detection crystal together with the probe beam. Figure 6(B) shows the time-domain signals for different THz field amplitudes for both free-space and crystalline quartz. It can be clearly seen that the time delay experienced by the pulse in the crystal quartz increases with the increase of the THz field amplitude, and the inset demonstrates the increase. Figure 6(C) and (D) show the nonlinear phase and absorption coefficient experienced by the signal of different amplitude at 0.4 THz, respectively, the value of the Kerr coefficient is calculated to be (9 ± 1.4) × 10^−14^ m^2^/W.

**Figure 7:**
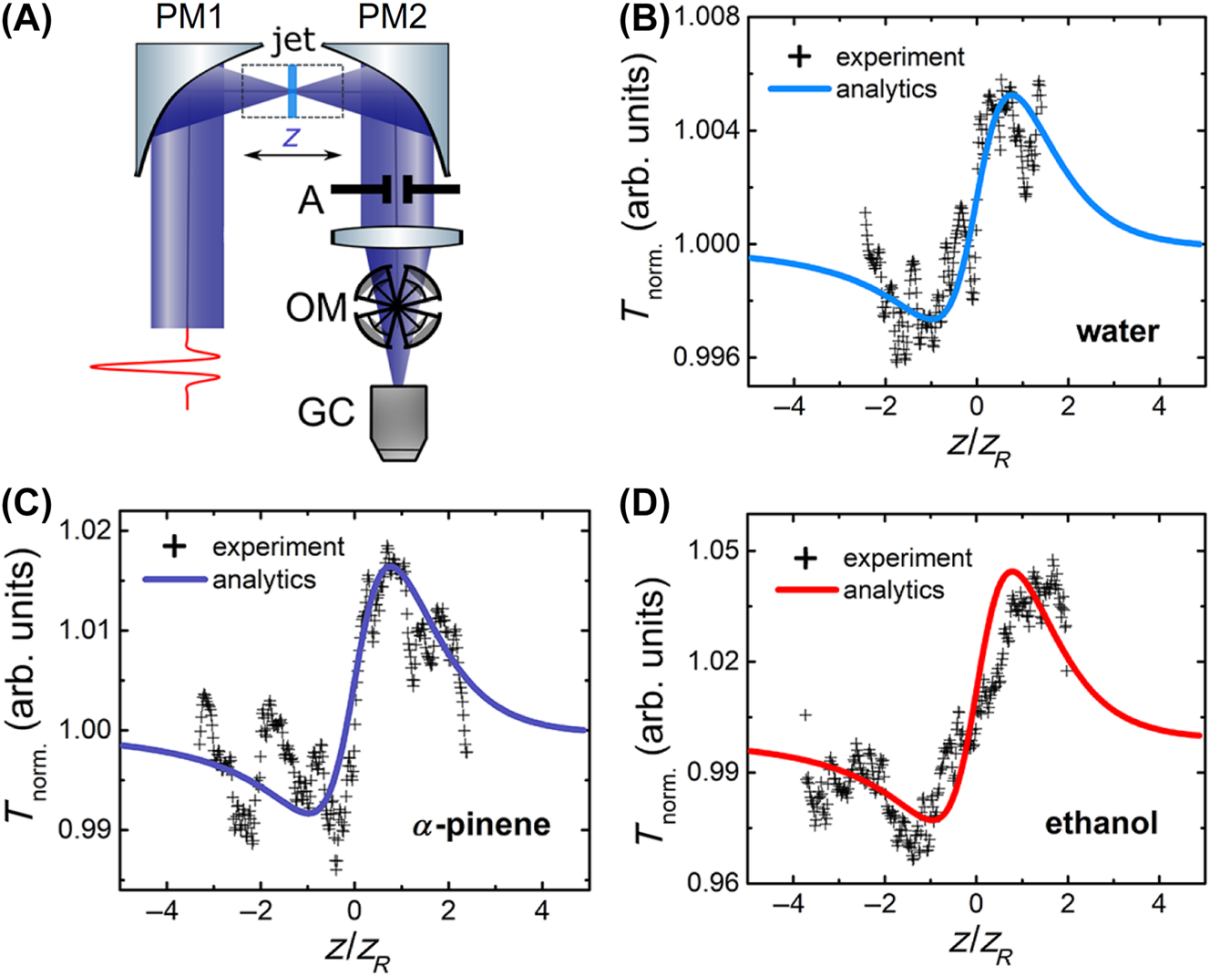
Strong Kerr responses in liquids [44]. (A) The schematic of the experimental setup for the *z*-scan measurements. The measured closed-aperture traces (crosses) and the analytical fits (solid lines) for (B) distilled water, (C) *α*-pinene, and (D) ethanol. Reprint permission obtained for references [44].

Liquids with noncentrosymmetric molecules, because of the vibrational response of these molecules, are reported to show giant Kerr coefficients in the THz region, which are as much as 6 orders of magnitude larger than their corresponding values in the visible or near-IR. As shown in Figure 7(A), the measurements are performed based on a *z*-scan technique [44]. The THz pulse, generated by optical rectification in a MgO:LiNbO_3_ crystal, is tightly focused onto the sample by mirror PM1 and then recollimated by parabolic mirror PM2. The sample is translated back and forth from the beam focus while measuring the transmitted THz pulse energies using a Golay cell with (without) an aperture A (transmittance of 2 %) to produce the closed (open) aperture traces, as shown in Figure 7(B)–(D). The experimentally measured nonlinear refractive index values of *n*_2_ in the THz regime are 7 × 10^−14^ m^2^/W for water, 3 × 10^−13^ m^2^/W for *α*-pinene, and 6 × 10^−13^ m^2^/W m^2^/W for ethanol. Since temperature exerts an influence on the motion of molecules in a liquid, the nonlinearity of the liquid can be modulated by temperature. Aleksandra et al. realized the control of the giant low-inertia nonlinear refractive index of water in the THz frequency range via temperature variation [45].

The *z*-scan technique is reported to be used in measuring nonlinear refractive index of other substances, for example, lactose and silicon [46]. Lactose, a kind of organic crystal, shows a large negative nonlinear refractive index, up to −1.49 × 10^−12^ cm^2^/W. It was found that Kerr nonlinearity can be enhanced in silicon at high THz intensities due to thermal effects or acceleration of carriers, up to 3.51 × 10^−12^ cm^2^/W.

The THz Kerr effect can manifest itself in many forms from which third-order nonlinearities can be extracted. The absorption at the position of resonances has been observed [47], which increases with the THz intensity, and the estimated value of the effective third-order susceptibility is extremely large, up to (0.4 + 6*i*) × 10^2^ m^2^/V^2^. The nonlinear THz transmission through subwavelength hole array in superconducting niobium nitride (NbN) film has been experimentally investigated using intense THz pulses, showing a significant change in dielectric constant [48]. The third-order nonlinearity of silicon is weak, but after being doped, it can become very strong, especially at the resonance [37], greatly improving the efficiency of four-wave mixing at THz regime. Metamaterials show giant Kerr coefficients, for example, it has reported that the resonant frequency can be changed [10], and the resonant mode can be shifted [49], through changing the amplitude of the input THz pulses.

In addition, the TKE is gradually moving toward applications, for example, the propagation of broadband THz pulses the influence of the Kerr effect has been investigated [50], and the propagation characteristics of surface Plasmon polaritons on a patterned graphene sheet incorporating a subwavelength ribbon resonator and a Kerr nonlinear bounding medium has also been studied [51].

### 4.2 Nonlinear THz birefringence

**Figure 8:**
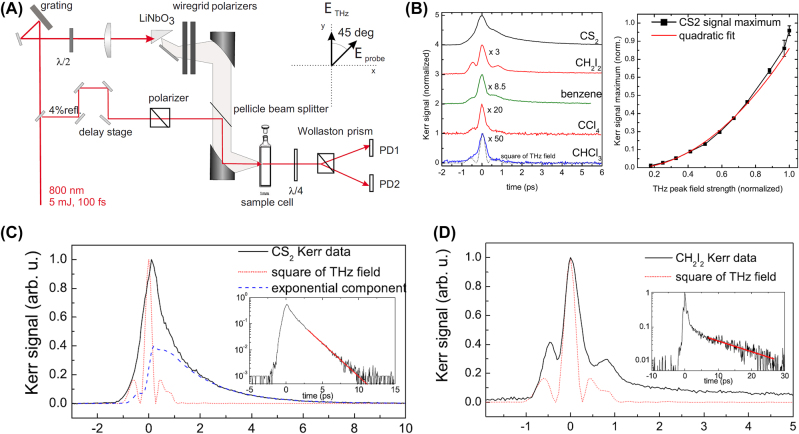
Optical birefringence in liquids induced by single-cycle THz pulses [41]. (A) Experimental setup. (B) THz Kerr signals obtained from five different liquids. The magnitude of the Kerr signal (shown for CS_2_) scales quadratically with the applied THz field. (C) and (D) The THz Kerr signal in CS_2_ and CH_2_I_2_, respectively. The insets show data on a log scale together with exponential fits yielding decay constants. Reprint permission obtained for references [41].

The refractive index at optical frequencies can not only be perturbed not by optical pulses but also be modulated by THz pulses. A representative work is the optical birefringence in liquids induced by single-cycle THz pulses [41], which is reported by Hoffmann et al. As shown in Figure 8(A), single-cycle THz pulses with energies exceeding 1.5 μJ were generated by the tilted pulse front technique and then were collimated and focused onto the sample where the THz intensity exceeded 50 MW/cm^2^, while a weak 800 nm probe beam was passed collinearly through the sample at a polarization of 45° with respect to the THz polarization. The THz-induced birefringence would change the polarization of weak probe and then was recorded through electro-optical sampling technology. As is shown in Figure 8(B), the Kerr signals for five liquids were measured. It is shown that the magnitude of the Kerr signal of CS_2_ scaled quadratically with the applied THz field, as expected as a *χ* ^(3)^ process. The measured values of nonlinear refractive index are on the same order of magnitude as values reported for all-optical measurements. Processes in the THz frequency range correspond to timescales on the order of molecular relaxation constants in liquids and glasses; they also observed higher-order nonlinear effects, that is, the inter- and intramolecular contributions to the Kerr signals. As shown in Figure 8(C) and (D), the Kerr signals remain after the THz pulses leave the samples, which correspond to orientational diffusion of molecules.

TKE has been reported for a variety of morphological substances. The instantaneous Kerr nonlinearity of air has been measured, showing the retarded alignment of molecules [46]. In order to study the effect of ionic concentration on water behavior in the ultrafast time scale, the TKE in deionized, distilled, and buffered water have been observed, showing that ions significantly alter water birefringence amplitude and its dynamics [52]. Similar tactics are utilized, it has been reported that first coherent excitation of intramolecular vibrational modes via the nonlinear interaction of a THz light field with molecular liquids [53] and the collective mode in the nematic superconducting state of Ba_1−*x*_K_*x*_Fe_2_As_2_ are also be reported to be driven by THz pulses [54]. Moreover, the ultrafast intermolecular hydrogen bond dynamics of water has been revealed by the TKE induced transient birefringence [55].

In terms of applications, THz pulses can enable the extreme laser spectral broadening [56] and generation of supercontinuum [57], as reported.

## 5 THz nonlinear absorption

**Figure 9:**
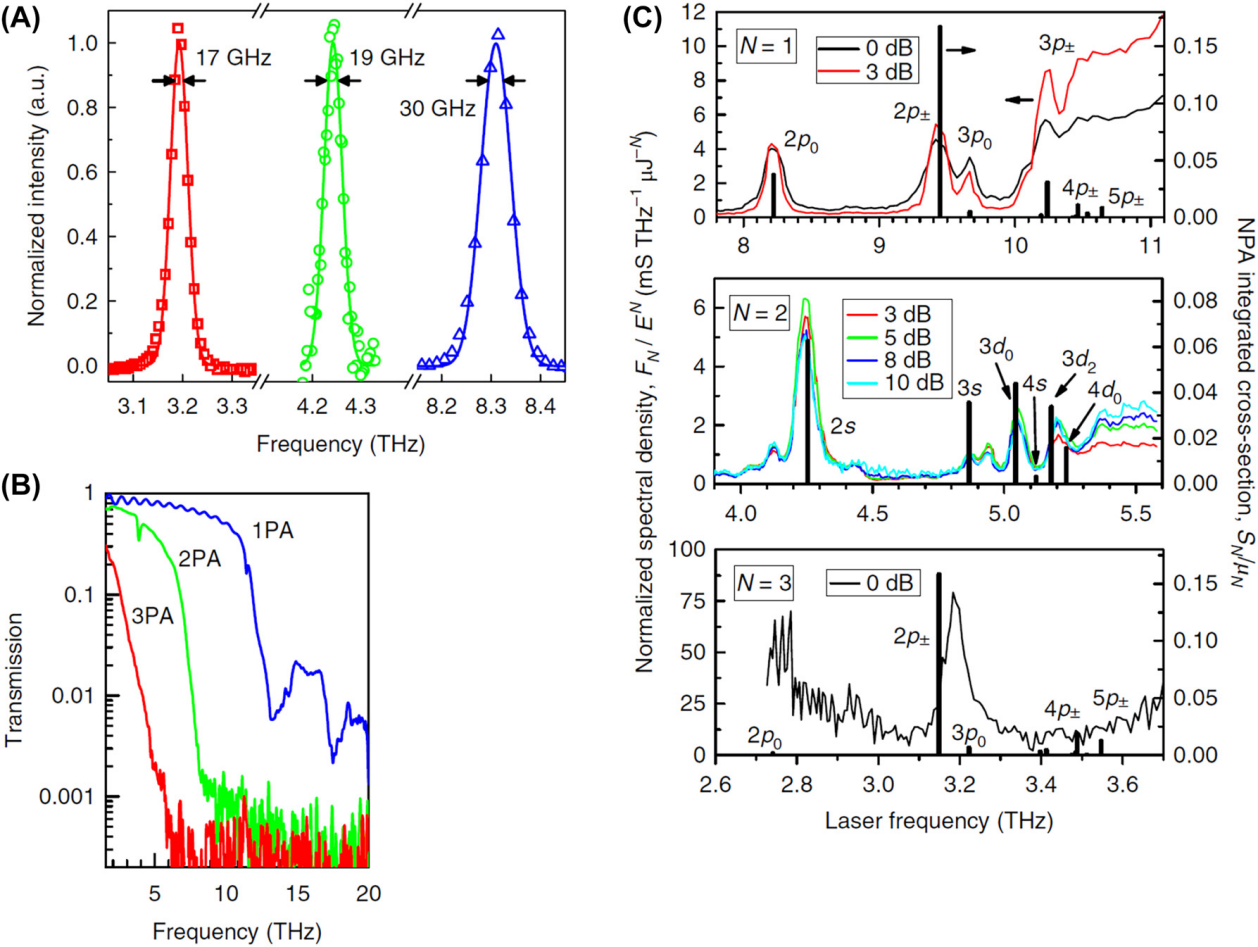
Multiphoton absorption of THz waves in semiconductors [58]. (A) Example experimental FELIX spectra (symbols) with different center frequencies, and the Gaussian fit is shown as a solid line; (B) spectra of filters used for each NPA experiment; (C) N-photon photoconductance spectra for various laser attenuations. Reprint permission obtained for references [58].

Multiphoton absorption is a well-known nonlinear process in atomic vapors, dyes, and semiconductors and finds applications in various photonic devices, such as autocorrelators, modulators, and sources of correlated photons. The rate of N-photon absorption (NPA) in semiconductors scales proportionally from hydrogen atoms, with factors of exponents 6N and 4N, indicating the potential for extremely large enhancements. In 2018, Loon et al. explored the nonlinear process of multiphoton absorption in silicon hydrogenic donors, revealing unprecedented absorption rates [58]. As shown in Figure 9, they observed 1-photon absorption (1PA), 2-photon absorption (2PA), and 3-photon absorption (3PA) in Si:P using a THz free-electron laser. The 2PA coefficient for 1s–2s at 4.25 THz was 400,000,000 GM, many orders of magnitude larger than in other systems. This research represents a significant step forward in the field of quantum control and THz technology. Its implications for advancing our understanding and capabilities in photonics are considerable, potentially leading to more efficient and powerful quantum devices. The study’s focus on silicon, a widely used semiconductor, makes its results particularly relevant and potentially transformative for a range of technological applications.

In the field of THz technology, the study of saturable absorption in a wide range of materials is providing groundbreaking insights with profound implications for future device development. Here, we explore the intricate interactions and responses of various materials, including graphene, silicon, InGaAs, GaAs/AlGaAs, and metamaterials, under the influence of THz fields.

**Figure 10:**
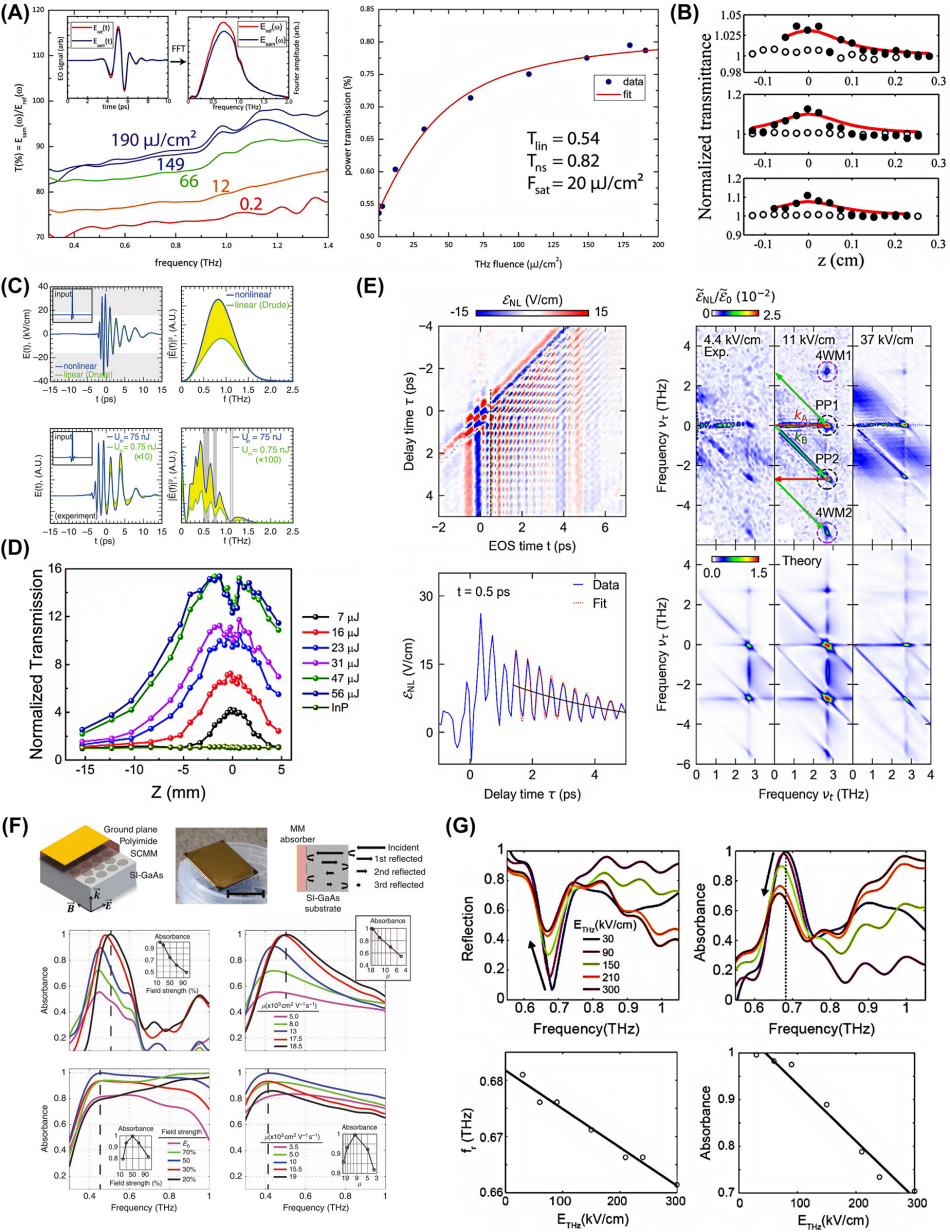
Researches on THz saturation absorption. (A) THz field transmittance of CVD graphene on fused silica at different fluences (left) and THz power transmission integrated over the entire THz pulse versus THz fluence (right) [59]; (B) *z*-scan traces of three samples (solid dots) and of the substrate (empty dots). The red lines are the fitting curves assuming the simple two-level saturable absorber model [60]; (C) calculated and measured transmitted THz waveform and power spectra. The gray bands show the absorption bands caused by water vapor in the ambient air, which causes additional absorption that was not included in the numerical simulation [61]; (D) *z*-scan normalized transmission of various THz pulse energies measured by the Golay cell. THz transmission values are normalized to the value measured at *z* = −15.3 mm with a THz pulse energy of 7 μJ [62]; (E) Fourier transform of ${\tilde{\varepsilon }}_{NL}$ time-domain data *ɛ*_NL_ for experimental field amplitudes of 4.4, 11, and 37 kV/cm, each normalized to the spectral amplitude ${\tilde{\varepsilon }}_{0}$ of the driving field at 2.7 THz [63]; (F) measurements for the saturation absorption with 18-μm-thick polyimide layer and optical limiter with 40-μm-thick polyimide layer, respectively. Insets show the absorbance trends as a function of field strength and electron mobility (*μ*) at the frequencies indicated by the dashed lines (mobility unit: ×10^3^ cm^2^/(V s) [64]; (G) measured reflection spectra of the nonlinear MPA under different THz field strengths and the absorbance at 0.68 THz versus the incident THz peak field [65]. Reprint permission obtained for references [59], [60], [61], [62], [63], [64], [65]. (B) Reproduced with permission [60], Optica Publishing Group. (C) Reproduced with permission [61], Optica Publishing Group. (D) Reproduced with permission [62], Optica Publishing Group. (E) Reproduced with permission [63], Optica Publishing Group.

Graphene is known for its saturable absorption properties in the near-infrared to visible spectrum. It also plays a significant role in the THz domain. In 2013, Hwang et al. discovered that CVD-grown graphene exhibits a pronounced THz-induced transparency [59], as shown in Figure 10(A). This phenomenon is attributed to the nonlinear pumping of carriers, which results in a suppression of carrier mobility and enhanced scattering processes. Therefore, graphene is an ideal candidate for ultrafast THz saturable absorbers. The validation of induced transparency in graphene is supported by time-resolved THz pump/THz probe spectroscopy. This indicates absorption recovery on a picosecond time scale, which is consistent with electron–hole recombination and carrier cooling. In 2015, Bianco et al. investigated the THz response of turbostratic multilayer graphene on silicon carbide at room temperature [60]. Figure 10(B) shows a significant 10 % improvement in transparency when subjected to strong THz fields. The saturation intensity is mainly attributed to the Pauli blocking effect in intrinsic graphene layers. The results suggest that controlling crystalline disorder and the number of layers can engineer THz nonlinear absorption properties, providing a promising platform for high-performance THz saturable absorbers and novel graphene-based mode-locked THz lasers.

In 2015, Li et al. explained the absorption of THz waves in silicon dielectric waveguides using a linear model of conduction [61]. However, under strong THz fields, saturation absorption is observed, necessitating a nonlinear dynamical model of Drude conductivity. As is shown in Figure 10(C), this model not only explains the saturation absorption phenomenon but also predicts field-induced transparency, attributing the nonlinear effect to velocity saturation, a fundamental limit on the speed of semiconductor devices. The implications of the study are significant for the design of future high-power THz guided-wave nonlinear devices, including frequency converters, parametric oscillators, mixers, and modulators.

In 2018, Chai et al. demonstrated the crossover from intraband to interband nonlinear optics at higher intensities in n-doped semiconductor InGaAs under intense THz pulses [62]. Figure 10(D) indicates an increase in low-energy free charge carriers and a subsequent reduction in THz transmission, making InGaAs an excellent candidate for a saturable absorber up to a certain THz field intensity. The study highlights the importance of considering interband effects and the time scale of strong absorption relative to the total pulse duration when designing and optimizing optical systems in the THz frequency range. In 2019, Raab et al. investigated intersubband (ISB) transitions in semiconductor multi-quantum well (MQW) structures as potential saturable absorbers at THz frequencies [63]. As shown in Figure 10(E), using amplitude- and phase-resolved two-dimensional (2D) THz spectroscopy on a subcycle timescale, the study observes clear signatures of THz-driven carrier-wave Rabi flopping. This investigation provides a deep understanding of the subcycle dynamics and paves the way for the design of customized MQW heterostructures implementing saturable absorbers in the THz range. Future quantum cascade laser (QCL) structures based on these findings may enable passive mode locking to generate high-power, widely tunable, ultrashort THz pulses from electrically biased semiconductor devices.

In 2016, Seren et al. discovered the strong nonlinear response of InAs plasmonic disc arrays in Figure 10(F). This was mainly due to electric field–induced interval scattering, which led to a reduction in carrier mobility and damping of the plasmonic response [64]. These arrays are used to create nonlinear perfect absorbers that can function as either optical limiters or saturable absorbers. Transferring these arrays to polyimide films allows for flexibility and conformal adhesion to curved surfaces, expanding their potential applications in ultrafast THz optics and as protective layers against strong resonant electromagnetic fields. As shown in Figure 10(G), Zhao et al. conducted a study on the nonlinear response of THz metamaterial perfect absorbers. These absorbers consist of electric split-ring resonators (ESRRs) on GaAs [65]. The nonlinear response of the absorbers is due to THz field-driven interband transitions and interband scattering in the GaAs. The ability to create flexible nonlinear absorbers has promising applications, including optical confinement and self-focusing. These metamaterials have the potential to achieve various functionalities such as saturable absorption, optical confinement, and self-focusing through careful structural design.

In summary, the saturation absorption of different materials under THz fields not only broadens our understanding of their physical properties but also provides a fundamental blueprint for the future of THz technology. This covers a range of applications from optical limiters to modulators and other nonlinear devices.

## 6 THz-induced IR/visible nonlinear effect

Considering the significant modulation of ionic nonlinearity by THz waves, it can be used to enhance the nonlinear coefficients of various optical materials in the visible and infrared bands, thereby regulating various nonlinear effects. In this section, we would like to introduce the nonlinear optical phenomena at visible or infrared induced by THz waves.

**Figure 11:**
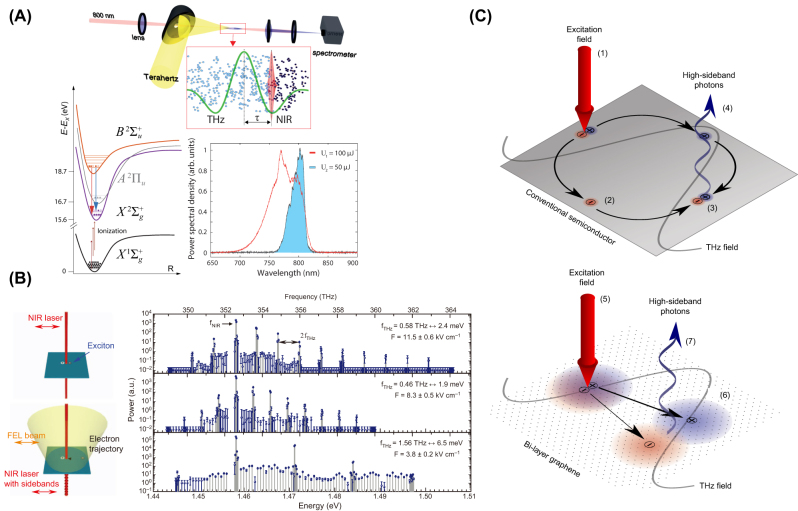
THz-induced harmonics in visible/infrared wavelengths. (A) Experiment design of the THz-driven coherent emission in molecular nitrogen ions [66]; (B) linearly polarized NIR light creates excitons when incident upon the quantum well sample and the experiment result [67]; (C) schematics of high-sideband generation in conventional semiconductors and in graphene [68]. Reprint permission obtained for references [66], [67], [68].

In early years, electric field–induced second-harmonic (EFISH) effect has attracted widespread attention as it can be used to achieve nonlinear response control of various media [16], [69], [70], [71], [72]. Due to the ability of THz waves to generate a very high instantaneous electric field in crystals, they have been widely used in recent years to replace the induction and control of SHG by external electric fields, namely THz field–induced second-harmonic (TFISH) generation. In comparison with EFISH, which drives the ions away from the original position by static electric field, TFISH triggers the ionic nonlinearity through a resonant way. The resonant excitation enables strong coupling between the input THz waves and optical phonons and thus to generate stimulated phonon polaritons. In this case, the ionic nonlinearity could be triggered to the uttermost. In 2015, Chen et al. proposed an EFISH of 800 nm laser by applying a DC bias voltage to a gold electrode on BiFeO_3_ thin films and achieved intensity modulation of the second harmonic by incident THz waves [73]. They also studied the effect of Sm doping concentration on this process in BiFeO_3_ thin films. In 2020, Bodrov et al. realized second-harmonic generation through TFISH in fused silica crystal by using laser pulse as excitation source and THz wave to provide strong driving field [74]. Afterward, Bilyk et al. generated an electric field of 23 MV/cm using THz waves and achieved similar effects on Ba_0.8_Sr_0.2_TiO_3_ thin films [75]. Additionally, THz waves can also achieve control over the nonlinear second-harmonic generation process in other systems, such as near-infrared laser pulses to irradiate nitrogen molecules and control the coherent emission process of nitrogen molecules by inducing second-harmonic generation using THz waves [66], as seen in Figure 11(A).

Similar to SHG, THz waves can also achieve the generation and regulation of high-order harmonics in infrared or visible light. Mao et al. theoretically studied the process of THz waves inducing high-order harmonic generation on graphene [79]. Li et al. generated high-order harmonics in ZnO thin films by simultaneously using near-infrared pulse excitation and THz wave modification fields, achieving conversion efficiencies of 5 % (6th) and 90 % (18th). They also observed suppression of odd order harmonics, proving that THz fields can be used to generate and regulate high-order harmonics in near-infrared light [80].

THz waves can also regulate the sideband high-order harmonic emission of semiconductor materials. In 2012, Zaks et al. used near-infrared laser to excite quantum wells to generate excitons and then removed electrons from the excitons using a strong THz field, causing them to collide with the generated holes again, resulting in high-order harmonic emission of the sidebands [67], shown in Figure 11(B). Cross et al. then theoretically predicted that using a weaker THz field can produce similar effects on graphene, schematically shown in Figure 11(C) [68].

**Figure 12:**
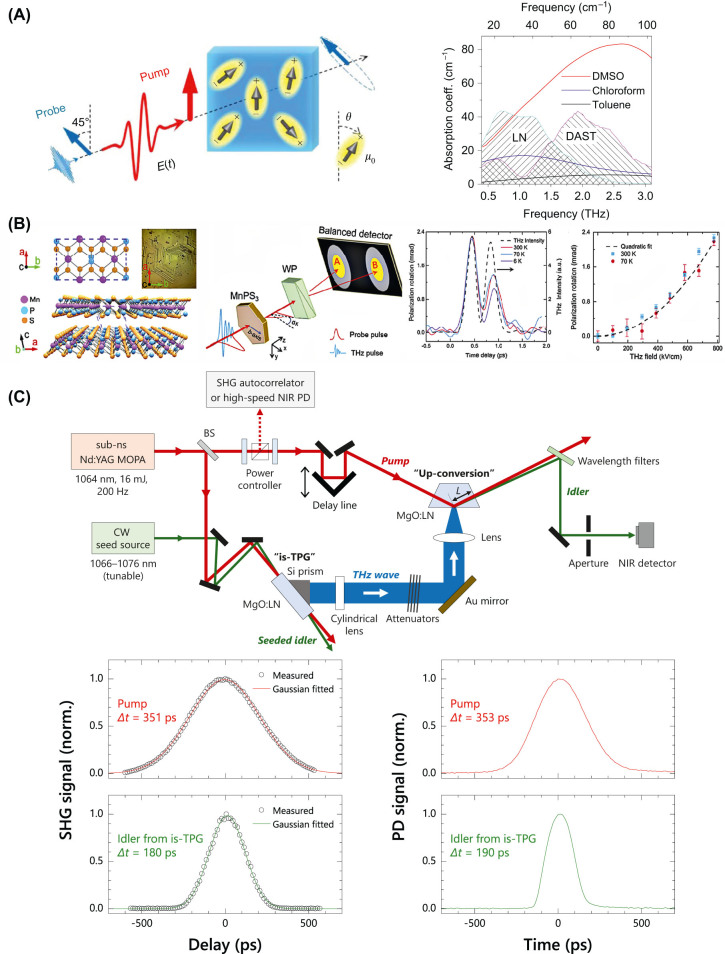
Terahertz-induced Kerr effect and nonlinear propagations. (A) Intense THz pulses induce birefringence in a polar liquid and the equilibrium THz absorption spectra of DMSO, chloroform, and toluene [76]; (B) lattice structure of MnPS_3_ single crystal and the experiment of nonlinear THz Kerr effect in quasi-2D MnPS_3_ [77]; (C) schematic experimental setups for the detection and characterization of THz wave pulses from the sub-ns injection-seeded THz-wave parametric generator (is-TPG) and NIR pulse characterization of pump and seeded idler from the is-TPG and the experiment result [78]. Two sets of experiment data measured SHG intensity autocorrelation traces and its Gaussian fitted curves and measured high-speed PD waveforms of pump and seeded idler from the is-TPG operated at 1.50 THz, respectively. Reprint permission obtained for references [76], [77], [78]. (B) Reproduced with permission [77], Optica Publishing Group. (C) Reproduced with permission [78], Optica Publishing Group.

Besides, THz waves are able to regulate the refractive index distribution inside crystals through nonlinear effects, thereby regulating the transmission properties of visible and near-infrared light in optical materials. In 2017, Vicario et al. studied the transmission behavior of 800 nm laser pulses in GaP crystals under THz wave modulation of optical properties [56]. They discovered the nonlinear modulation of crystals by THz waves, which includes two aspects: THz wave–induced Kerr effect and Pockels effect. Sajadi et al. studied the resonant nonlinear excitation of polar liquids (dimethyl sulfoxide, DMSO) by THz waves and found that THz waves enhance the optical birefringence of liquids, which is one order of magnitude higher than visible light enhancement [76], show in Figure 12(A). In 2019, Giorgianni et al. induced time-varying optical phase modulation of organic crystal OHQ-N2S using THz waves to generate supercontinuum spectra in the near-infrared band, achieving spectral broadening of 280 nm and controllable wavelength shift [57].

The Kerr nonlinearity of many materials can be controlled using THz waves, including solid liquids and gases, crystals and amorphous, bulk materials and two-dimensional materials, etc. Zalkovskij et al. investigated THz wave–induced Kerr nonlinearity in amorphous chalcogenide glasses As_2_S_3_ and As_2_Se_3_ [40]. For a near-infrared pulse of 800 nm, *n*_2_ = 1.746 × 10^−14^ cm^2^/W (As_2_S_3_) and *n*_2_ = 3.440 × 10^−14^ cm^2^/W (As_2_Se_3_) were measured. Shalaby et al. investigated the THz wave–induced Kerr nonlinearity of carbon dioxide, nitrogen, and oxygen [81]. Sajadi measured the Kerr nonlinearity of materials such as diamond, sapphire, magnesium oxide (MgO), polymethylpentene (TPX), low-density polyethylene (LDPE), silicon nitride film (SiN), and crystalline quartz induced by THz waves by studying optical birefringence [82]. Cheng et al. investigated the refractive index anisotropy of quasi two-dimensional material MnPS_3_ induced by THz waves and measured its Kerr nonlinearity, as seen in Figure 12(B) [77].

In addition, THz waves were also used to induce frequency up-conversion of near-infrared pulses in trapezoidal MgO: LN crystals and indirectly measured the time-domain signal of sub-nanosecond THz wave pulses by measuring near-infrared lasers, as shown in Figure 12(C) [78].

## 7 Conclusions

In this review, we reported on the critical progresses of THz nonlinear phenomena, with focusing on both THz frequency-conversion processes, THz Kerr nonlinearity, nonlinear absorption, as well as THz-induced nonlinearity on visible or infrared frequencies. These achievements are based on crystalline materials, low-dimensional materials, or even in liquids, and all of them represent the recent progress in the field of THz nonlinear optical physics. Benefit from the high-power THz sources, THz nonlinearities have achieved considerable progress. The selection of the spectral range and field amplitude is critical to obtain giant THz nonlinearity. Generally, the spectral range of the THz source could benefit the THz nonlinearity if it is close to the eigenfrequency of optical phonons, by which the strong nonlinearity of stimulated phonon polaritons can be better utilized. Besides, the power intensity of the THz source should not be too strong in case that the nonlinear materials could be damaged.

After involving the phonons and proposing the stimulated phonon polaritons, THz waves, acting as one of the most essential modulation approaches to crystal lattice, depict a promising blueprint in polariton studies, material science, and nonlinear control over polar crystal properties in thermal optics, optomechanics, spin, illumination, and ferroelectricity/ferromagnetism. As an important quasiparticle not relying on cryogenic environment, stimulated phonon polaritons, excited by THz waves, may be expected to own a very broad research blank in the following decades. Actually, many researchers have performed very broad material modulation through THz waves, while they are less possible to be included in this review for the page limit.

All reported progresses here represent the state of the art, not yet fully exploited, and the fertilizing ground of the nonlinear light–matter interaction at THz frequencies. In the near future, considerable exciting nonlinear investigations on THz nonlinear physics would arise, accompanied by the gradually developed high-power THz sources.
